# The Importance of High-Quality Cataract Surgery: A Case of Objective Improvement in Post-surgery Outcomes at a Rural Hospital in Vietnam

**DOI:** 10.7759/cureus.93673

**Published:** 2025-10-01

**Authors:** Eisuke Shimizu, Hiroki Nishimura, Rohan J Khemlani, Tadashi Hattori, Hiroshi Fujishima

**Affiliations:** 1 Ophthalmology, Yokohama Keiai Eye Clinic, Kanagawa, JPN; 2 Department of Ophthalmology, Keio University School of Medicine, Tokyo, JPN; 3 Ophthalmology, OUI Inc., Tokyo, JPN; 4 Ophthalmology, Non-Profit Organization, Fight For Vision, Tokyo, JPN; 5 Ophthalmology, Non-Profit Organization, Asia Prevention of Blindness Association, Kyoto, JPN; 6 Department of Ophthalmology, Tsurumi University School of Dental Medicine, Kanagawa, JPN

**Keywords:** cataract, eye camp, optometry, smart eye camera, vietnam

## Abstract

The Socialist Republic of Vietnam, with a population of approximately 100 million, faces a critical shortage of ophthalmologists with only 1,300 practitioners nationwide (1.3 per 100,000 population). Cataracts remain the leading cause of blindness, accounting for over 75% of cases. While volunteer cataract surgery programs operate throughout the country, resource limitations often preclude comprehensive pre- and post-operative assessments, including standardized visual function testing. We report a case from a rural Vietnamese hospital, demonstrating significant objective visual improvement after cataract surgery, which highlights both the challenges and the public health significance of surgical outreach in resource-limited settings.

An 86-year-old Vietnamese woman presented with vision loss and no documented medical or ophthalmic history. She underwent volunteer-based cataract surgery on her right eye. Preoperative findings included uncorrected visual acuity of 0.03 (20/667), intraocular pressure (IOP) of 13.0 mmHg, and a dense nuclear sclerotic cataract (grade 4-5) precluding fundoscopic examination. Phacoemulsification with intraocular lens (IOL) implantation was performed without complications. Postoperative management included topical antibiotics and overnight patching. On postoperative day one, the patient’s uncorrected visual acuity improved to 0.6 (20/33). IOP was 14.0 mmHg, and findings included mild anterior chamber inflammation, well-centered IOL, and a clear fundus view with normal macula and optic disc morphology.

This case highlights that cataract surgery in a rural, low-resource setting can result in substantial visual improvement, even in advanced cases. It emphasizes cataracts as a leading cause of reversible blindness in Vietnam and demonstrates the transformative impact of surgical outreach. Expanding access and training are essential to address unmet ophthalmic needs in underserved regions.

## Introduction

Cataracts remain the leading cause of global blindness, especially in low- and middle-income countries [1]. This burden disproportionately affects developing countries, where limited access to surgery leads to preventable vision loss, making cataracts a primary target for blindness prevention initiatives [2]. In Vietnam, cataracts have historically dominated blindness statistics. Previous studies revealed that 25% of post-cataract patients achieved corrected visual acuity below 6/24, indicating suboptimal outcomes [3]. Rural and mountainous areas show higher prevalence due to limited access compared to urban centers [4]. The 2015 Vietnam National Blindness Survey attributed 74% of blindness to cataracts. An aging population and surgical backlog have intensified this public health challenge [5].

While economic development has increased the prevalence of diabetic retinopathy and glaucoma, common in developed nations, Vietnam faces unique challenges: inadequate postoperative compliance, limited rural healthcare, and the dual burden of communicable and non-communicable diseases [1,6]. These factors underscore the critical importance of outreach surgical programs in underserved communities [5]. This report describes our experience with a volunteer cataract surgery program in northern Vietnam, documenting visual recovery in an elderly female patient with near-complete preoperative vision loss.

## Case presentation

The case

**Figure 1:**
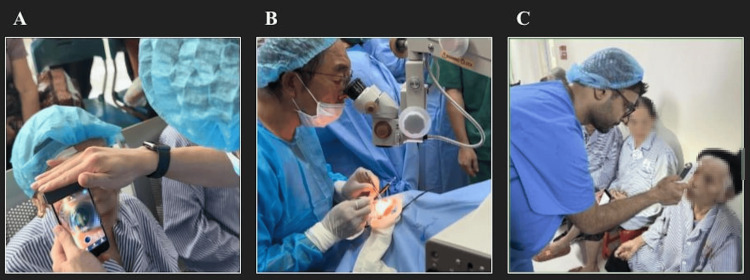
Volunteer cataract surgery workflow. (A) Pre-op using portable slit-lamp. (B) Intraoperative cataract extraction. (C) Day 1 post-op intraocular pressure assessment with portable tonometry.

An 86-year-old woman from rural northern Vietnam presented with bilateral vision loss and no documented ophthalmic history. According to her family members, she had experienced complete vision loss in her left eye approximately 10 years prior, but did not seek medical attention as her right eye remained functional. Progressive vision loss in her right eye over the past five years has significantly impaired her activities of daily living. Geographic isolation and economic constraints prevented access to ophthalmic care until presentation at a volunteer surgical outreach program (Figure 1).

Clinical findings at pre-surgical examination (just before the surgery)

**Figure 2:**
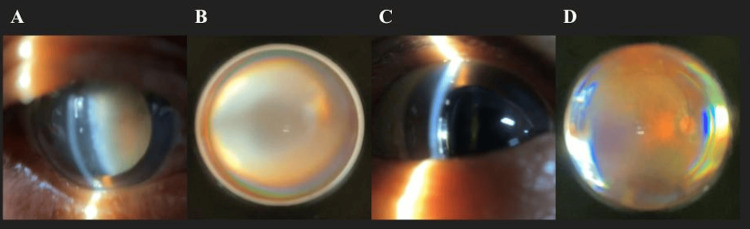
Ocular findings pre- and post-surgery (A) Grade 4-5 nuclear cataract pre-op. (B) Fundus obscured by lens opacity. (C) Day 1 post-op: clear cornea, well-positioned IOL. (D) Post-op fundus: normal disc and retinal vasculature.

The Initial examination revealed an uncorrected visual acuity of 0.03 (20/667) in the right eye and no light perception in the left eye. Pupillary light reflex was present only in the right eye. Intraocular pressure was measured at 13.0 mmHg in the right eye and 18.0 mmHg in the left eye using a handheld tonometer (iCare) [7,8]. A portable slit-lamp microscope demonstrated a mature cataract in the right eye that precluded fundus examination (Figure 2). The cornea was clear with normal anterior chamber depth bilaterally, and no other significant anterior segment abnormalities were noted.

Surgical intervention

Phacoemulsification with posterior chamber intraocular lens (IOL) implantation was performed on the right eye under retrobulbar anesthesia. Due to poor red reflex, 0.06% trypan blue was utilized for anterior capsule visualization during continuous curvilinear capsulorhexis. The advanced nuclear sclerosis initially prompted consideration of extracapsular cataract extraction; however, phacoemulsification was successfully completed without conversion and any complications. A +20.0 diopter posterior chamber IOL (Model RF-22L) was implanted in the capsular bag. The IOL power was calculated using the SRK/T formula based on the axial length measured by A-mode (ultrasound) and the corneal curvature radius measured with an autorefractor keratometer. Intracameral and topical antibiotics (ofloxacin 0.3% ointment and levofloxacin 1.5% drops) were administered at procedure completion. The procedure was performed by two skilled ophthalmologists (N. DTK, and C. HV).

Postoperative day 1 examination

First-day postoperative examination demonstrated remarkable visual improvement with uncorrected visual acuity of 0.6 (20/33) in the right eye, representing an increase from baseline. Intraocular pressure remained stable at 14.0 mmHg. Anterior segment examination revealed a mild cellular reaction in the anterior chamber (grade 1+) with a clear cornea showing no edema and a well-apposed surgical wound. The clear ocular media now permitted fundoscopic examination, which showed normal macular and optic disc morphology (Figure 2). No other postoperative complications were observed.

## Discussion

This case demonstrates successful visual rehabilitation through volunteer-based cataract surgery in a resource-limited setting, with visual acuity improving objectively from 0.03 (20/667) to 0.6 (20/33). Volunteer surgical missions in remote areas face significant logistical challenges [9]. For example, our program must transport complete surgical infrastructure, including phacoemulsification units, operating microscopes, intraocular lenses, pharmaceuticals, and consumables. Pre- and postoperative assessment capabilities are often sacrificed due to transport limitations. Moreover, our surgical site exemplifies these challenges, lacking a dedicated ophthalmology department and located in a mountainous region requiring four to six hours of travel from Hanoi despite being only 200 kilometers away. Even basic standard equipment, such as stationary slit-lamp biomicroscopes, could not be transported due to road conditions. Our report identifies critical success factors and operational challenges encountered in volunteer cataract surgery programs [9].

Our site is one of Vietnam's poorest regions, where patients often lack access to routine ophthalmic care and face financial constraints that prevent them from following up at specialty centers. This underscores the value of portable diagnostics [10]. Our ability to perform comprehensive examinations using portable devices transformed what traditionally relies on subjective patient reports ("I can see better") into objective, measurable outcomes.

While advanced cataracts often yield suboptimal surgical results due to associated complications, our case achieved excellent visual recovery [11]. This success is particularly meaningful given that cataract-related blindness remains endemic in rural Vietnam, where ophthalmic resource scarcity perpetuates the disease burden [12]. The profound impact of restored vision on quality of life has been well-documented in Vietnamese populations, with studies demonstrating significant improvements in vision-related quality of life scores following cataract surgery.

This case illustrates that even mature cataracts can achieve good outcomes with appropriate surgical technique and perioperative care. Such documented successes serve dual purposes: providing immediate patient benefit and demonstrating to local communities the transformative potential of cataract surgery. These visible outcomes may help overcome cultural and economic barriers to seeking ophthalmic care.

However, this report has several limitations. Follow-up was restricted to the first postoperative day, and only a single case was examined, so long-term outcomes remain unknown. These factors may underestimate potential risks, such as posterior capsule opacification or challenges with patient adherence. As a result, the findings should be interpreted cautiously, and their generalizability is limited.

Moving forward, sustainable models for volunteer surgical programs should prioritize: (1) incorporation of portable diagnostic equipment for objective outcome assessment, (2) partnerships with local healthcare systems to establish follow-up protocols, and (3) community education to promote earlier presentation. Our continued engagement in Vietnam aims to address the substantial backlog of cataract blindness while building local capacity for ongoing care.

## Conclusions

This case demonstrates that high-quality cataract surgery in rural, low-resource settings can achieve excellent visual outcomes, even in advanced cases. In a region with limited access to routine ophthalmic care, the integration of portable diagnostic tools allowed objective assessment of surgical success and helped bridge the gap left by the absence of stationary ophthalmic equipment. The profound improvement in visual acuity, from 0.03 (20/667) to 0.6 (20/33), not only restored the patient’s functional independence but also served as a tangible example to the community of the benefits of surgical intervention. Such visible results may help overcome cultural hesitations and economic barriers that often delay presentation for treatment.

Beyond individual patient impact, this experience underscores the broader public health implications of addressing cataract-related blindness in underserved areas. Sustainable expansion of outreach programs will require coordinated efforts to improve surgical logistics, incorporate portable diagnostic technologies, and strengthen local healthcare partnerships to ensure follow-up care. Training and empowering local providers, coupled with targeted community education, can promote earlier detection and intervention, ultimately reducing the burden of avoidable blindness in rural Vietnam and similar settings.
